# The *ProActive* trial protocol – a randomised controlled trial of the efficacy of a family-based, domiciliary intervention programme to increase physical activity among individuals at high risk of diabetes [ISRCTN61323766]

**DOI:** 10.1186/1471-2458-4-48

**Published:** 2004-10-18

**Authors:** Kate Williams, A Toby Prevost, Simon Griffin, Wendy Hardeman, William Hollingworth, David Spiegelhalter, Stephen Sutton, Ulf Ekelund, Nicholas Wareham, Ann Louise Kinmonth

**Affiliations:** 1 General Practice and Primary Care Research Unit, Department of Public Health and Primary Care, Institute of Public Health, Forvie Site, Robinson Way, Cambridge CB2 2SR, UK; 2 Department of Radiology, 146 N Canal St, Suite 300, University of Washington, Seattle, WA, 98103, Box 358853, USA; 3 MRC Biostatistics Unit, Institute of Public Health, Forvie Site, Robinson Way, Cambridge CB2 2SR, UK; 4 MRC Epidemiology Unit, Strangeway Research Laboratory, Wort's Causeway, Cambridge CB1 8RN, UK

## Abstract

**Background:**

Increasing prevalence of obesity and disorders associated with sedentary living constitute a major global public health problem. While previous evaluations of interventions to increase physical activity have involved communities or individuals with established disease, less attention has been given to interventions for individuals at risk of disease.

**Methods/design:**

***ProActive ***aims to evaluate the efficacy of a theoretical, evidence- and family-based intervention programme to increase physical activity in a sedentary population, defined as being at-risk through having a parental family history of diabetes. Primary care diabetes or family history registers were used to recruit 365 individuals aged 30–50 years, screened for activity level. Participants were assigned by central randomisation to three intervention programmes: brief written advice (comparison group), or a psychologically based behavioural change programme, delivered either by telephone (distance group) or face-to-face in the family home over one year. The protocol-driven intervention programme is delivered by trained facilitators, and aims to support increases in physical activity through the introduction and facilitation of a range of self-regulatory skills (e.g. goal setting). The primary outcome is daytime energy expenditure and its ratio to resting energy expenditure, measured at baseline and one year using individually calibrated heart rate monitoring. Secondary measures include self-report of individual and family activity, psychological mediators of behaviour change, physiological and biochemical correlates, acceptability, and costs, measured at baseline, six months and one year. The primary intention to treat analysis will compare groups at one-year post randomisation. Estimation of the impact on diabetes incidence will be modelled using data from a parallel ten-year cohort study using similar measures.

**Discussion:**

***ProActive ***is the first efficacy trial of an intervention programme to promote physical activity in a defined high-risk group accessible through primary care. The intervention programme is based on psychological theory and evidence; it introduces and facilitates the use of self-regulatory skills to support behaviour change and maintenance. The trial addresses a range of methodological weaknesses in the field by careful specification and quality assurance of the intervention programme, precise characterisation of participants, year-long follow-up and objective measurement of physical activity. Due to report in 2005, ***ProActive ***will provide estimates of the extent to which this approach could assist at-risk groups who could benefit from changes in behaviours affecting health, and inform future pragmatic trials.

## Background

This trial addresses the rise in the burden of disease associated with sedentary living: a major public health problem. Physical inactivity accounts for up to 11.7% of all deaths in developed countries [1] and it has been causally associated with coronary heart disease, diabetes, osteoporosis and some cancers. The rise in the prevalence of obesity in many countries may be associated with a decline in physical activity.

Reversal of this trend will require not only public health programmes to increase activity at societal level, but also interventions to help high-risk individuals increase physical activity and maintain beneficial activity patterns [2]. This is a trial of such an intervention. It aims to overcome the limitations of previous studies through careful choice and characterisation of the target population, study design, measures, and the interventions under evaluation themselves. The intervention programme, based on theories and evidence from psychology about how best to support behavioural change and maintenance, is potentially generalisable to other settings, target groups and behaviours.

### Target population

The study targets people with a parental family history of Type 2 diabetes and a sedentary lifestyle, who constitute a clearly identifiable high-risk population [3]. A consistent direct relationship exists between sedentary living and Type 2 diabetes [3,4]. People with a family history of diabetes have a three-fold increased risk of developing diabetes compared to those without; a risk that is magnified by physical inactivity and weight gain [3,5]. At least 40% of the excess risk associated with weight gain might be avoided in such people if their BMI did not exceed 30 kg/m^2 ^[2,3], and prospective studies support the idea that physical activity reduces weight gain [5,6].

### Limitations of previous trials

Most trials have evaluated increasing physical activity in the context of established disease. The few published trials of primary prevention in high-risk groups have methodological limitations. They have mainly evaluated brief interventions to increase exercise in the general population delivered through primary care practitioners [7-9], often with very short follow-up (a few weeks). Those such as the Activity Counselling Trial ("ACT"), offering follow-up for two years, are based on an unknown proportion of willing attendees at ambulatory care facilities [10,11]. Evaluation of exercise prescriptions delivered through leisure centres has not been encouraging [12]. Moreover, participants have been poorly characterised in terms of risk, and most studies have relied on self-reports of exercise. This may inflate differences between groups due to recall bias, and cannot capture changes in either total energy expenditure, or physical activity related energy expenditure [13]. Measuring total energy expenditure is relevant if an increase in one component of activity results in a decline in another, as activities are substituted, and measuring physical activity related energy expenditure (i.e. total energy expenditure adjusted for resting energy expenditure) is important if this is the aetiologically relevant factor.

Three trials among individuals with impaired glucose tolerance in China [14], Finland [15] and USA [16], have established that intensive approaches to lifestyle changes including physical activity can delay progression to diabetes by 58% over three years [15,16] and possibly longer [14]. Few studies have modelled the long-term effects of physical activity, although available work suggests that interventions for primary prevention of Type 2 diabetes might be cost-effective [17]. A full review of primary prevention trials [5] identified no interventions aimed at increasing physical activity alone, without accompanying dietary change or intended weight loss. The current study addresses this gap, and previous methodological shortcomings, by careful characterisation of the participants, year-long follow-up, objective measurement of physical activity, and modelling of the relationship between current behaviour change and future disease risk.

### Limitations of previous interventions

#### (i) Poorly specified interventions

Many of the available trials evaluated interventions that were not explicitly based on psychological theory and evidence, and did not specify clearly which behaviour change techniques were applied by the providers [18-20]. In addition, interventions often used relatively ineffective behaviour change techniques, for instance giving people advice about behaviour change [21,22]. More effective interventions to promote physical activity have applied psychological theory and evidence about how best to support behaviour change [16,23]. The development of the ***ProActive ***intervention programme included a review of psychological theories and evidence, through systematic reviews [18,20] and expert meetings, and a one-year feasibility study among 15 willing participants and their families. Based on this work, the Theory of Planned Behaviour (TPB) [24] was selected as the theoretical framework to inform behavioural determinants targeted in the intervention. Determinants include beliefs and attitudes towards the behaviour (here physical activity), which are elicited at an individual level. Tailoring interventions to personal beliefs is an innovative, but theoretically appropriate application of the TPB. Systematic reviews and expert meetings then informed the selection of potentially effective techniques aimed at changing beliefs. They include reinforcement of positive beliefs, and problem solving in relation to negative beliefs, in order to strengthen motivation.

#### (ii) Attention to adoption and maintenance of physical activity

A range of behaviour change techniques with evidence for their effectiveness was used to bridge the gap between intention and action: goal setting and review, action planning, use of prompts, self-monitoring, and reinforcement [2,18,23,25]. Use of a causal model, linking measured beliefs and attitudes to behaviour will allow subsequent process analysis to better specify both determinants and intervention (Hardeman *et al*., 2004. A causal modelling approach to the development of theory-based behaviour change programmes for trial evaluation. Submitted).

Major challenges in promoting physical activity are maintenance of behaviour change, and the avoidance of drop out rates that can approach 50% [5,26]. Reviews suggest that theoretical advances in facilitating behaviour maintenance have not been applied in intervention programmes [27,28]. The highest levels of participation have been achieved by home-based interventions, involving frequent professional contact, and promoting enjoyable, informal exercise of moderate intensity, such as walking [2,8]. Behavioural maintenance may be best supported by building habits, using self-regulatory strategies such as repetition of behaviours over time in a constant environment, ongoing goal review, self-monitoring, reinforcement, and relapse prevention [28,29]. The use of mail and telephone contacts is a promising cost-effective approach [23], especially the use of frequent, brief, support calls [2]. All these approaches are incorporated into ***ProActive***.

### ProActive objectives

The primary objective of ***ProActive ***is to determine the effects of a theoretical- and evidence-based intervention programme on objectively measured physical activity after one year, in sedentary individuals at risk of diabetes and related metabolic abnormalities due to their family history. Three questions are posed:

1. Behaviour change: Can an innovative approach to increasing physical activity achieve clinically important change in this behaviour when offered to a group at increased risk of diabetes?

2. Disease impact: If so, what is the potential for the changes in behaviour achieved in mid-life to reduce the incidence of diabetes in later life?

3. Dose finding: How does delivery of the approach, at two levels of intensity, affect acceptability, efficacy and costs?

The trial will estimate the extent to which physical activity and its key psychological mediators are altered by the intervention programme, and assess its acceptability to this high-risk group. It will document the extent to which behaviour change is associated with reduction in weight gain and improvement in physiological and biochemical correlates, and will model the potential impact of the intervention on future risk of diabetes.

Intensive, face-to-face interventions may not be a feasible health service model, and there is some evidence that less intensive, continuous support may be as effective [2,23]. The intervention programme is therefore being evaluated at two levels of intensity: 'face-to-face' (delivered at the participants' homes and by telephone) and 'distance' (delivered by one home visit and telephone and correspondence) over one year, in order to inform the most cost-effective intervention programme for wider evaluation. If potential efficacy is demonstrated, we intend to proceed to a multi-centre pragmatic trial of the cost-effectiveness of the approach in practice.

## Methods/design

***ProActive ***is a four-year study with a complex randomised trial design [30], with central randomisation of willing participants to intervention programmes or comparison. The trial is managed from the Institute of Public Health, University of Cambridge, following MRC guidelines. Ethical approval has been obtained from the Eastern MREC, and West Suffolk, Cambridge, Huntingdon and West Essex LRECs.

**Figure 1:**
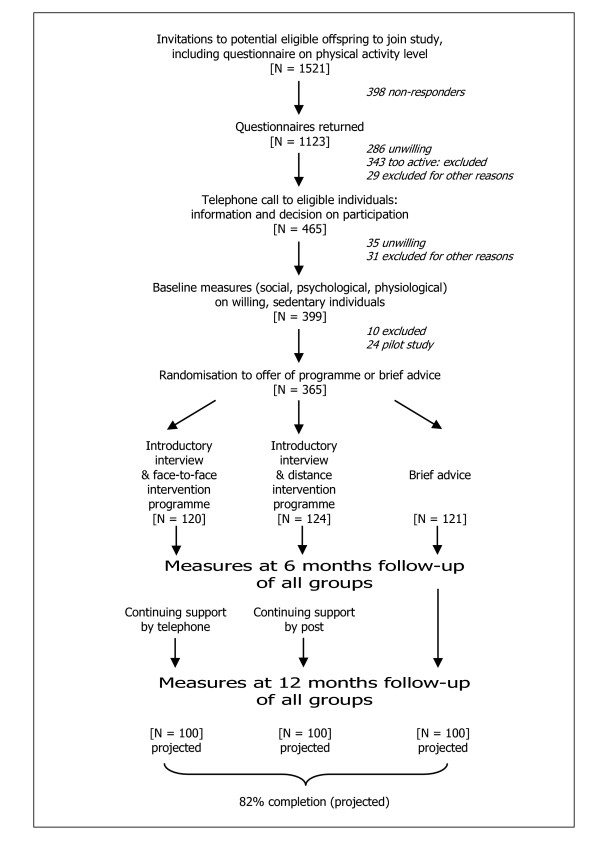
Trial design and patient flows; Oct 2003 (recruitment closure)

The study design and patient flows (achieved at recruitment closure, October 2003, and projected to end of study) are shown in Figure 1. The focus of measurement is the adult offspring of a Type 2 diabetic parent, but the focus of the intervention programme is this individual within a family context. Participants were recruited via parents with diabetes on primary care registers (20 practices), or directly through records of their family history of diabetes (seven of the 20 practices). Sedentary individuals and their families were randomised to facilitation, either 'face-to-face' or 'distance', or to a comparison arm offering a leaflet providing brief advice on the benefits of activity. Psychological, physiological, anthropometric and biochemical data were collected at baseline and one year after randomisation, with psychological data also collected at six months after randomisation.

The study is explanatory in design, and the quality-assured intervention programmes are delivered by carefully trained and supervised family health facilitators with experience of working in primary care or the community, and backgrounds in health promotion, dietetics and nursing.

### Setting, recruitment and screening

The study is set in urban, suburban and rural Cambridgeshire, Essex and West Suffolk, England, in the homes of participants and their families. The study population consists of offspring of people with Type 2 diabetes, aged 30–50 years, without a diagnosis of diabetes, and not considered very active based on self-report at the start of the study (see below). This age range defines a group at risk of weight gain [31,32]. Any individuals found at study entry to have fasting hyperglycaemia [33] were referred to their family doctor, but retained in the trial.

### Practice recruitment

Once the relevant ethical and PCT approval had been obtained, 53 practice teams in the locality were approached by letter, inviting them to take part in the study, and highlighting the reimbursement of all costs involved. Personalised letters were sent to the practice manager (who we asked to collate responses and reply using a reply slip and Freepost envelope), all partners and nursing staff. Included with each letter was a brief summary of the study and a Research Information Sheet for Practices (RISP) form [34]. If no response was received, a follow-up phone call was made to the practice manager. A principal investigator and member of the trial team visited interested practice teams, to discuss the study in further detail. All relevant practice staff were encouraged to attend, particularly those who would be involved in the administration of proposed patient surveys. The 20 practices that agreed to take part then received a 'set-up' visit by the trial team. A 'Practice Survey Manual' was created for the practice staff, and the trial team supported the practice teams as needed throughout the survey period.

### Participant recruitment

Initially participants were recruited through their parents; patients with Type 2 diabetes on the diabetes registers of 20 practices ('recruitment method 1'). Patients were written to by their general practitioner, with a description of the study, and asked to provide contact information for any offspring aged 30–50 years, living locally. Consent was also sought for the practice to pass the contact details of the offspring to the research team so that they could invite the offspring directly into the study. Piloting demonstrated feasibility and acceptability of the method, and one reminder was sent after three weeks if no reply was received.

From 20 practices, 2631 patients were approached and 2025 (77%) replied, yielding 1238 potentially eligible offspring who were invited to take part in the study. The ratio of approximately one potentially eligible offspring to two patients with diabetes was half our pilot projections, so to increase recruitment we developed a second recruitment approach ('recruitment method 2'). This approach recruited potential participants with a recorded family history of diabetes directly from practices with family history registers, and was feasible in seven of the 20 practices. General practitioners wrote to all patients aged 30–50 years with a recorded family history of diabetes, enclosing a study information sheet, and asking those willing to complete and return to the practice a questionnaire to determine which family member(s) had diabetes, and of which type. Consent was sought for this information and contact details to be passed on to the research team. Using this method, with again one reminder letter, 1340 patients were written to, and 896 (67%) responses were received, with 283 patients interested and eligible. Both recruitment approaches provided 1521 potential trial participants. Practitioners used their discretion in applying both approaches to the exclusion of patients who were physically or mentally unwell.

### Study population: inclusion and exclusion criteria

#### Activity levels

Potential participants recruited by both methods were next written to by the research team with full information about the study and a screening activity questionnaire, describing occupational and leisure activity, based on published questionnaires [35,36], to exclude very active individuals. Two reminder letters with questionnaires were sent at two-week intervals if necessary, giving a response rate of 74%. Respondents were excluded if they reported their occupational activity as 'heavy manual work' [35]; or 'physical work' if their total score on the leisure questionnaire [36] was ≥ 20; or 'sedentary' or 'standing' work if their total leisure activity score was ≥ 30. This resulted in exclusion of approximately 30% of those screened, a figure that matches well with the proportion of the UK population designated as active in prevalence surveys [37].

#### Study requirements

To fulfil measurement requirements participants had to be able to walk briskly, without help, on the flat for 15 minutes. Participants also had to live within reach of the measurement centre and the Family Health Facilitators; defined as a 30-minute average travel time from the study co-ordination centre. Other exclusion criteria included individuals with serious physical or psychiatric illness limiting programme involvement; people with life issues interfering with the study; those known to be pregnant or have diabetes before baseline measurement; and those planning to move away. As shown in Figure 1, application of these criteria reduced the 837 'interested' responses to 465 potentially eligible individuals, who were telephoned by a trained interviewer to confirm eligibility. Eligible and interested individuals were then scheduled for baseline measurement at either the Ely Research Centre or the Addenbrooke's Hospital Wellcome Trust Clinical Research Facility, where written consent was obtained.

Eligible offspring were registered with general practitioners in the Eastern Region of the UK. Prior to both baseline measures and randomisation, these doctors were individually informed about their registered patients' intention to participate in ***ProActive***. Brief details of the trial were sent, together with a request for feedback if the practitioner had any concerns about the offspring's participation, or about the safety of the facilitators making home visits.

### Randomisation

Randomisation was carried out centrally by the trial statistician, using a partial minimisation procedure that dynamically adjusted the randomisation probabilities in order to balance important covariates; body mass index, sex, age, physical activity (individually calibrated heart rate monitoring, see below), family size, and behavioural intentions. Randomisation thus used baseline measures. Thirty-two pairs of siblings and two sibling-triples were cluster randomised to the same study group to avoid contamination, and the remaining 295 participants (81%) were individually randomised. Overall, 365/465 (78%) of those eligible went forward to randomisation.

### Baseline measures and follow-up

At baseline and the end of the study, all participants attend the study centre at either Ely or Cambridge for questionnaires, physiological and anthropometrical measures, and venesection. At six months, psychological and self-reported physical activity data are collected by postal questionnaires. Measures relating to the intervention programme evaluation are collected by the facilitators during the intervention, and we assess reported use of self-regulatory strategies by participants to increase their activity levels at six and twelve months.

### Compliance with follow-up

In similar primary care based trials we have achieved attrition rates of 30% or less [38,39], and at current rates we will exceed the required 300 to complete the study (100 in each group, see Figure 1).

Maximising retention is an important issue, particularly as the comparison group do not benefit from regular contact with a facilitator. At recruitment, the introductory leaflets for all three arms emphasised the importance of follow-up, irrespective of treatment group. Participants who drop out of the intervention programme are contacted by a principal investigator, and offered an opportunity to give feedback and to confirm drop out from the intervention programme only, or from trial measurement as well.

### Measurement

The distribution of measures across baseline, six-month and one-year follow-up are shown in Table 1. The principle outcome is an objective measurement of physical activity energy expenditure, the daytime physical activity ratio (dayPAR), which is the ratio of daytime energy expenditure to resting energy expenditure measured using heart rate monitoring with individual calibration for the heart rate-energy expenditure relationship [40,41]. This allows more precise quantification of the relationship between energy expenditure and relevant disease end points than self-report [13]. The method has been validated against the gold standard techniques of doubly-labelled water and whole-body calorimetry [42]. Physical activity is also measured by a validated questionnaire covering work, recreation and domestic activity over the previous month and year [43], and offspring report of usual physical activity patterns among family members and how they changed over the previous year.

**Table 1:** Study measures

**Measures**	**Baseline**	**6 months**	**12 months**
**Questionnaire measures:**			
1. Godin / EPIC self-reported physical activity [36]			
2. Short form State anxiety [45]			
3. Risk / worry diabetes^+^			
4. Theory of Planned Behaviour [24]^+^			
5. General Questionnaire, comprising:			
A) Rose Angina questionnaire			
B) Smoking, Alcohol & Physical Activity^+^			**(smoking &** **physical activity ** **only)**
C) Occupation & Social Class			
D) SF-36 & EQ-5D [44,46]			
6. EPAQ (2) [43]			
7. Clinical measurement questionnaire (physiological measures and family history)			
8. Physical activity of family members^+^			
9. Injury questionnaire^+^			
10. Intervention programme satisfaction^+^*			
11. Skills acquisition^+^*			
**Physiological measures: **[13,40-42,51]			
Cardiorespiratory fitness & dayPAR parameters			
Weight, height, % body fat, blood pressure, ECG			
Biochemical parameters (fasting plasma glucose, glycosylated haemoglobin, insulin, lipids)			
Blood stored for future genetic testing			
**Costs:**			
Cost to the NHS of facilitator training & salary^+^			
Costs of intervention programme delivery^+^			

^+ ^questionnaires developed for study

* intervention programme participants only

Oxygen uptake (ml O_2_/kg/body weight) is measured by indirect calorimetry during a submaximal graded treadmill exercise test, and maximal cardiorespiratory fitness (VO_2max_) is estimated using predicted maximal heart rate (i.e. 220 minus age) [40,42].

Self-report measures of well-being and quality of life include subjective health and energy (SF-36) [44], anxiety [45], worry about diabetes and perceived vulnerability, and EuroQol (EQ-5D) [46].

The frequency and severity of physical activity related injury is assessed by study questionnaire at one year.

Psychological mediators of physical activity include intention to increase activity over the next year, and its predictors (attitude, subjective norm, perceived behavioural control). These key measures have been developed for the study following the recommendations of Ajzen [24]. Physiological correlates of behaviour include weight measured on standard scales calibrated at three monthly intervals, body fat percentage measured by bio-electrical impedance (Bodystat, Isle of Man, UK), and systolic/diastolic blood pressure, measured using an automatic sphygmomanometer (Accutorr, UK). Biochemical correlates include fasting plasma glucose, glycosylated haemoglobin, insulin and lipids. We are storing EDTA whole blood samples for future genetic testing. Sociodemographic factors and ECG are also documented at baseline.

### Cost of the intervention

The economic analysis will explore the impact of a physical activity intervention programme on NHS costs. As the study is explanatory in design, we will not conduct a full cost-effectiveness analysis, but aim to provide a cost-description of the delivery of the intervention programmes. We are measuring the costs of delivering the 'face-to-face' and 'distance' intervention programmes via family health facilitators. These costs primarily comprise the training of facilitators, educational materials, travel, and the time that facilitators spend contacting and visiting families (including cancelled visits). Travel costs and contact time are recorded by the facilitators for every trial participant. The cost of facilitator time will be based on national average salaries, employment costs, qualifications, overheads and indirect costs [47]. Although we do not expect the ***ProActive ***intervention programme to have an impact on health service costs in the short term, we are monitoring health service utilisation (hospital, primary and community care) in the last 20% of participants recruited to the study.

### Participant safety

The primary safety concerns for participants in ***ProActive ***are cardiovascular and musculoskeletal events associated with the laboratory procedures of treadmill exercise testing and injuries sustained as a consequence of increasing physical activity in everyday life. The cardiorespiratory fitness test used in this study is submaximal, and only undertaken following extensive screening procedures. If a participant exhibits a positive Rose angina questionnaire [48], a positive physical activity readiness questionnaire [49] or an abnormal ECG, they are referred to a clinical member of the measurement team for a more detailed medical review. If there are clinical concerns, participants are excluded from the study, and referred to their general practitioner. In over 3000 such tests undertaken by our group using this protocol, no significant adverse events have occurred. Supervising staff are trained and hold current cardio pulmonary resuscitation certificates.

Ranges for acceptable results are set for all clinical measures. If these are exceeded, the information is sent to the general practitioner, and the participant informed and advised to consult.

As the intervention programme is based on participants' own preferred activities, and emphasises small achievable goals set by the participants, the risk of excess injury is small. Group information about injury will be reported.

Participants previously unaware of their familial risk of diabetes may experience anxiety related to awareness of their increased risk status. This is considered in facilitator training, and measures of anxiety, worry about diabetes and perceived vulnerability are included (see above).

### Data management, quality assurance and exclusion of bias

Physiological and anthropometric measures are made in two centres by observers unaware of individuals' group allocation. Biochemical measures are made in one laboratory with established quality assurance systems. Randomisation was undertaken by the trial statistician, independently of the trial co-ordination team, and the data entry team are unaware of study group.

The administrative database (participant information), dayPAR values and blood test results are managed in-house, with the latter being double entered. Numeric fields have limiters set so that values outside a defined range cannot be entered. Additionally, any blood results outside the 'normal range' are flagged for confirmation of value. Random checks on administrative data are performed regularly, checking the data on the database against paper records and correcting any errors found.

Double data entry of all anthropometric and questionnaire measures is undertaken by an experienced, independent agency, blind to study group (Wyman Dillon Research and Data Management, Bristol, UK). In addition, random checks are applied as described above.

### Intervention (see Figure 1)

#### Intervention programme contacts

The family health facilitator contacts participants randomised to the 'face-to-face' and 'distance' interventions, and arranges a home interview including family members. At this introductory interview, personal reasons for increasing physical activity are elicited and reinforced, family participation is encouraged, and the relationships between physical activity, weight gain and prevention of Type 2 diabetes are explained and discussed.

In the 'face-to-face' arm this is followed by four visits and two brief support telephone calls over five months. During these interactions the participant and willing family members learn strategies to increase physical activity, for instance selecting activities that they enjoy doing, setting achievable goals, defining action plans, self-monitoring, self-reinforcement and relapse prevention. Pedometers are available for self-monitoring among participants who have chosen walking as their goal. A key difference between this intervention and others currently under evaluation (e.g. ACT) is that there is no absolute target for physical activity defined at the outset. Family members are encouraged to make gradual and continuous increases in their activity, as much as they feel able to, on the understanding that all increases, if maintained, are beneficial. Follow-up continues by monthly telephone calls up to one year, to discuss any difficulties in applying the strategies, and to encourage family members to increase activity further.

In the 'distance' arm, following the introductory meeting the intervention programme is delivered by six telephone calls over five months, and then monthly by post up to one year, with content similar to the 'face-to-face' arm. During the phone calls the facilitators encourage the participants to involve family members. Visits and telephone calls take approximately one hour and 45 minutes, respectively.

#### Materials

An arm-specific introductory leaflet is used, but otherwise materials are the same for the face-to-face and distance arms. All introductory leaflets include text to encourage retention in the trial. In the comparison arm the leaflets offer brief advice on the benefits of physical activity. Participants in the intervention programme arms are given an educational manual describing the strategies that participants are encouraged to use to increase their habitual activity in a step by step fashion.

#### Promotion of fidelity of intervention delivery

Various mechanisms are used to promote the fidelity of delivery of the intervention programme to the underlying psychological theories and intervention programme protocols. A detailed training manual and protocols for each contact were developed, and a Training Officer appointed. Facilitators attended a five day phased course in psychological theories, behaviour change techniques and experiential training in techniques, with six half-days initially, followed by refresher sessions at six months and continuing supervised practice by a clinical psychologist and through peer-appraisal. Facilitators complete a checklist for the introduction of and mastery of self-regulatory strategies by the participant after each contact, and monitor intervention programme attendance and drop-out for each participant.

#### Assessment of fidelity and evaluation of the intervention programme

An assessment of adherence by facilitators to the behaviour change techniques specified in the protocols was conducted among a random sample of 27 participants, using reliable coding frames and transcripts of the sessions. The intervention programme evaluation includes: an assessment of the frequency of meetings and telephone calls, proportion of progress reports and postcards sent and progress reports returned, satisfaction with the intervention programme, reported use of self-regulatory strategies by participants at six months and one year, and drop-outs at one year.

## Statistical procedures

### Sample size

The sample size calculation was initially based on physical activity level (PAL), the ratio of total energy expenditure to estimated basal metabolic rate [40,41], and required 100 individuals completing one-year follow-up in each group. Prior to the measurement of any follow-up data, and endorsed by the Trial Steering Committee, a proposal was made to change the primary outcome measure to dayPAR, the ratio of daytime energy expenditure to resting energy expenditure, on grounds that this outcome consisted entirely of measured rather than estimated quantities. The calculations were based on the Ely cohort study data [41,50], in which the residual standard deviation of one-year change in dayPAR adjusting for baseline was 0.53. With 100 individuals in each group, there is 80% power to detect a difference in mean dayPAR of 0.18 between the combined intervention programme groups and the control group with a two-sided test at the 5% level of significance. This is equivalent to 2 MET hours/day, 30 minutes of brisk walking on the level, or 20 minutes of leisurely bicycling or swimming; a plausible and important increase. The observed difference in mean dayPAR between any pair of groups will be estimated with a 95% confidence interval having the width ± 0.15, equivalent to ± 1.75 MET hours/day, ± 25 minutes brisk walking or ± 15 minutes bicycling or swimming. Calculations were based on equal numbers in each group, and require 300 participants with outcome data at one-year follow-up. Recruitment of 400 participants allowed for 25% attrition after randomisation, and lower interim attrition rates will enable a lower recruitment target of 365 participants (Figure 1).

Main analyses will be at one year, comparing combined 'face-to-face' and 'distance' versions of the intervention programme with 'brief advice', comparing 'face-to-face' with 'distance' modes of the intervention programme, and estimating the difference between each intervention programme group and 'brief advice' to inform a larger pragmatic trial. Analysis by intention-to-treat will retain individuals within their randomised group regardless of participation. Comparisons will involve an adjustment for baseline physical activity and other variables used in the randomisation. We will undertake sensitivity analyses, assuming a range of potential outcomes for non-completers, informed by available baseline and interim data on non-completers. Non-completers will have multiple data imputed with a 'missing at random' assumption and with sensitivity analyses to represent optimistic and pessimistic scenarios for drop out. Clustering effects by family will be estimated for the primary outcome.

A secondary 'dose-response' analysis will use all three randomised groups, over baseline, six months and one year. A 'per protocol' analysis will also be undertaken among those completing the intervention programme.

The incremental cost of delivering the 'face-to-face' intervention programme will be compared to the 'distance' and 'brief advice' groups.

### Modelling will comprise a series of stages

*Stage 1) *The trial will provide evidence on the relationship between observed behaviour change, weight change, and biochemical and physiological correlates. Modelling is facilitated by reference to the Ely Cohort; a prospective population cohort study that began in 1990 and involved 1122 people without known diabetes [40]. Measurements identical to those used in the Ely Cohort Study are included in ***ProActive***.

*Stage 2) *Using models based on past cohort data, the influence of behaviour change on future diabetes incidence [40,41,51,52] will be projected, appropriately allowing for uncertainty in the parameter estimates. Simulation methods will be adopted. At this stage other risk factors (e.g. smoking, diet) will be assumed fixed.

*Stage 3) *We will undertake sensitivity analyses on the projections at Stage 2, using a range of plausible assumptions about how behaviour change might affect other risk factors and hence indirectly influence future diabetes risk.

## Discussion

***ProActive ***is the first efficacy trial of physical activity promotion in a defined high-risk group accessible through primary care, evaluating an intervention programme based on theory and evidence. It supports increases in informal activity, through the introduction and facilitation of self-regulatory strategies with regular reinforcement by the facilitator. Due to report in 2005, ***ProActive ***has the potential to make substantial contributions to understanding the extent to which such approaches could assist the wide range of at risk groups who could benefit most from increasing their physical activity.

The trial team brings together expertise in the epidemiology of diabetes [53] with intervention development and evaluation [18-21,30], measurement from beliefs to self-reported behaviour [26] and objectively measured energy expenditure [13,40,54] and trials [55], especially in primary care [38,39]. Their complementary contributions will allow both the answering of the main study questions in a robust manner, and the development of theory and method for future studies. Further exploratory work on interactions between genotype, social class and physical activity are planned, which may in the future lead to refinement in selection of the at-risk group. As an adjunct to the measurement of physical activity related energy expenditure by individually calibrated heart rate monitoring, we are also employing measurement of body movement using the MTI-Actigraph [54] on a proportion of the participants. The combination of the two measurement techniques has the potential to overcome the limitations with either method used alone, and improves the estimates of physical activity related energy expenditure [56], since the measurement errors associated with the methods are not positively correlated.

In terms of the intervention itself, careful measurement along the hypothesised causal path from cognition, through self-reported behaviours to energy expenditure, will enable testing of the application of the Theory of Planned Behaviour in this setting, and of the relationship between the everyday activities that the programme has as its focus and the objectively measured physical activity (Hardeman *et al*., 2004. A causal modelling approach to the development of theory-based behaviour change programmes for trial evaluation. Submitted). This will enable replication and further strengthening of effective intervention steps, as well as development of theory.

Together, it is expected that the findings will inform the design of future larger scale and more pragmatic preventive programmes promoting physical activity in at-risk groups.

## List of abbreviations

dayPAR = daytime physical activity ratio; the ratio of daytime energy expenditure to resting metabolic rate measured using heart rate monitoring with individual calibration

ECG = electrocardiogram

MET = metabolic equivalent

PAL = physical activity level; the ratio of total energy expenditure to estimated basal metabolic rate measured using heart rate monitoring with individual calibration

TPB = Theory of Planned Behaviour

VO_2max _= maximal oxygen uptake (ml O_2_/kg/min)

## Competing interests

The authors declare that they have no competing interests.

## Authors' contributions

ALK, NW, SG, SS, WH, DS, – Principal Investigators

TP – Trial Statistician

KW – Trial Co-ordinator

Will H – Trial Economist

UE – Physical activity measurement

All authors read and approved the final manuscript. ALK is the paper guarantor.

## Pre-publication history

The pre-publication history for this paper can be accessed here:


